# Preliminary clinical and structural outcomes of a standardized ultrasound-guided diagnostic and interventional protocol for refractory adhesive capsulitis

**DOI:** 10.3389/fmed.2026.1872504

**Published:** 2026-09-15

**Authors:** Tamara L. Rodríguez-Araya, Tiago Mota Gomes, Anna Arias Gassol, Xavier Torres, Luciano Polino, Claudia Arango Silva, Helena Florez, Juan C. Sarmiento-Monroy, Ana Belén Azuaga, Virginia Ruiz-Esquide, Andrés Ponce, Andrea Zacarías Crovato, Beatriz Frade Sosa, Oriana Omaña, Georgina Novell Soms, Joshua Peñafiel-Sam, Isaac Pomés López

**Affiliations:** 1 Primary Chronic Pain Unit, Department of Rheumatology, Institute ICEMEQ, Hospital Clínic, Barcelona, Spain; 2 CAP Cassanova, Institute CAPSBE, Hospital Clínic, Barcelona, Spain; 3 Clinical Health Psychology Section, Department of Psychiatry, Institute ICN, Hospital Clínic, Barcelona, Spain; 4 Department of Rheumatology, Institute ICEMEQ, Hospital Clínic, Barcelona, Spain; 5 Musculoskeletal Ultrasound Section, Department of Radiology, Institute CDI, Hospital Clínic, Barcelona, Spain

**Keywords:** adhesive capsulitis, capsular thickness, frozen shoulder, hydrodilatation, shoulder pain, suprascapular nerve block, ultrasound-guided intervention

## Abstract

**Introduction:**

Adhesive capsulitis is common and often prolonged condition characterized by pain and progressive restriction of glenohumeral motion, with a substantial proportion of patients experiencing persistent symptoms and functional limitations. Despite the wide range of available treatments, clinical outcomes remain variable, reflecting heterogeneity in both diagnostic approaches and therapeutic strategies.

**Objective:**

To describe the short-term clinical and sonographic evolution following a standardized ultrasound guided diagnostic and interventional pathway combining suprascapular nerve block and glenohumeral hydrodilatation in patients with refractory adhesive capsulitis

**Methods:**

This study is a retrospective analysis of a prospectively maintained clinical database of patients managed within a standardized care pathway in a tertiary musculoskeletal interventional unit between December 2023 and April 2024. Patients with clinically and sonographically confirmed refractory adhesive capsulitis who underwent the combined protocol were included. Pain intensity, shoulder range of motion, and ultrasound-measured capsular thickness were recorded at baseline and at 3-months. Routine clinical follow-up was available for up to 12 months.

**Results:**

16 patients with confirmed adhesive capsulitis underwent the combined intervention. At 3 months, pain scores and range-of-motion measurements had improved compared with baseline, and ultrasound assessment demonstrated a reduction in inferior glenohumeral capsular thickness. No major procedural complications were documented. During routine clinical follow-up to 12 months, no recurrence was documented; one patient had persistent residual symptoms attributed to concomitant calcific tendinopathy.

**Conclusion:**

In this small uncontrolled cohort, a standardized ultrasound-guided pathway was followed by clinically meaningful improvements in pain and shoulder mobility and by a reduction in capsular thickness at 3 months. The parallel clinical and sonographic changes observed in this refractory population support further investigation of this integrated pathway. Although causality and the independent contribution of individual procedural components cannot be established, these preliminary findings provide a rationale for prospective controlled evaluation with blinded outcome assessment.

## 1 Introduction

Adhesive capsulitis (AC), also known as frozen shoulder or arthrofibrosis, is a disabling disorder of the glenohumeral joint characterized by progressive shoulder pain accompanied by a marked reduction in both active and passive range of motion (ROM). Despite being widely recognized in clinical practice, the exact etiology and pathophysiological mechanisms underlying this condition remain incompletely understood (1). AC affects approximately 2%–5% of the general population and is more common in women between 40 and 60 years of age, being relatively uncommon in individuals older than 70 years.

It is traditionally classified into primary (idiopathic) and secondary forms, the latter occurring in association with identifiable systemic or local conditions that may contribute to the development of capsular inflammation and subsequent fibrosis. Among local factors, prolonged shoulder immobilization, trauma, and previous surgery have been consistently associated with an increased risk of developing AC (1, 2). Among systemic conditions, diabetes mellitus represents the most consistently reported association, showing prevalence rates of up to 20% and has been linked to more severe symptoms and a more prolonged clinical course (1–3). Other reported associations include thyroid disorders, particularly hypothyroidism, cardiovascular and cerebrovascular diseases, autoimmune disorders, and Dupuytren’s disease (1–5). A potential genetic predisposition has also been suggested, with reported associations with family history and human leukocyte antigen B27 (HLA-B27) positivity (1, 2).

From a biological perspective, AC is characterized by a progressive transition from an initial inflammatory synovitis to a fibroproliferative capsular contracture, driven by increased expression of pro-inflammatory cytokines (IL-1, IL-6, IL-8, TNF-α), and fibrogenic mediators such as transforming growth factor-β (TGF-β) and platelet-derived growth factor (PDGF). These processes promote fibroblast proliferation, extracellular matrix deposition, and capsular thickening, ultimately leading to reduced capsular compliance and mechanical restriction of glenohumeral motion (1–3, 6). From a therapeutic perspective, this combined pathophysiological mechanisms provides a rationale for minimally invasive interventions targeting inflammation, pain and capsular stiffness. Intra-articular corticosteroids are widely used to modulate the inflammatory component of the disease, suprascapular nerve block (SSNB) may reduce nociceptive input and improve pain-related functional limitation, while capsular hydrodilatation (HD) aims to mechanically distend the contracted capsule, potentially restoring joint volume and improving mobility (1, 2, 7).

This condition typically presents with an insidious onset of shoulder pain followed by progressive stiffness, resulting in marked limitation of both active and passive ROM, particularly in external rotation. Pain is often poorly localized, may worsen at night and usually precedes the development of substantial motion loss. Classically, the condition has been divided into three clinical stages: a painful or “freezing” phase, characterized by increasing pain and progressive restriction of motion; a “frozen” phase, in which pain may gradually diminish but stiffness becomes more established; and a “thawing” phase, marked by slow and often incomplete recovery of shoulder mobility. Although AC has traditionally been described as a self-limiting disorder with spontaneous resolution within 1–2 years, long-term follow-up studies have shown that recovery is not always complete. Persistent pain or stiffness has been reported in up to 40% of patients, and residual limitation in ROM may remain in approximately 7%–15% of cases, although severe long-term functional disability is relatively uncommon (1, 2, 7).

Frozen shoulder remains primarily a clinical diagnosis and is often considered a diagnosis of exclusion (1, 8). Several shoulder disorders, including rotator cuff pathology, glenohumeral osteoarthritis, cervical radiculopathy, and other musculoskeletal conditions, may present with similar symptoms of pain and restricted ROM, making diagnostic differentiation challenging (2, 7, 8). Previous studies have highlighted the potential for diagnostic inaccuracies in patients referred with suspected frozen shoulder, suggesting that misdiagnosis is not uncommon in routine clinical practice (1, 2, 7–9). These findings underline the importance of careful clinical assessment and appropriate imaging when necessary to establish an accurate diagnosis.

Among available imaging modalities, ultrasound has emerged as a useful tool for evaluating capsular abnormalities and excluding alternative shoulder pathologies. However, despite its accessibility and dynamic capabilities, its role in the diagnostic assessment of AC remains underutilized in routine clinical practice. Given the pathophysiological relevance of capsular thickening, ultrasound assessment of capsular thickness may improve diagnostic accuracy and facilitate appropriate patient selection for interventional treatment, potentially reducing misdiagnosis and optimizing therapeutic outcomes (10–14).

Various therapeutic strategies have been proposed for the management of AC, including physiotherapy, oral anti-inflammatory medication, corticosteroid injections, manipulation under anesthesia and arthroscopic capsular release. However, these treatments may result in persistent pain and limited ROM in up to 50% of patients Nevertheless, conservative treatment approaches may fail to achieve satisfactory clinical improvement in up to 50% of patients (1, 2, 7, 8).

Hydrodilatation of the glenohumeral joint capsule has emerged as a minimally invasive therapeutic option aimed at mechanically distending the contracted capsule, thereby improving capsular compliance and shoulder mobility. Several studies have reported improvements in pain and ROM following this procedure (15, 16). However, reported outcomes vary substantially across studies, likely reflecting differences in procedural techniques and patient selection rather than the intrinsic effectiveness of HD itself. Key variables include injected volume, corticosteroid use, imaging guidance, injection approach, and whether capsular distension or rupture is achieved (15–20). In addition, inconsistent diagnostic criteria and inclusion of patients without clearly defined AC may further contribute to variability in results (15–17). These limitations highlight the need for a standardized diagnostic and interventional approach that ensures accurate patient selection and consistent application of image-guided techniques.

In addition to capsular distension techniques, regional nerve blocks represent a well-established therapeutic strategy in AC, with substantial evidence supporting their effectiveness in pain control. The suprascapular nerve provides approximately 70% of the sensory innervation of the shoulder joint and has therefore been widely targeted in the management of shoulder pain (21). Several studies have demonstrated that SSNB alone can produce significant improvements in shoulder pain and ROM in AC by reducing nociceptive input and facilitating participation in rehabilitation (22, 23). Given the combined role of pain and capsular contracture in limiting shoulder mobility, these approaches may be complementary rather than alternative. Effective analgesia may facilitate capsular mobilization, while mechanical distension techniques such as HD address the structural component of the disease. Accordingly, SSNB has been incorporated into interventional protocols to improve procedural tolerance and potentially enhance functional outcomes when combined with HD (18, 21–23).

In this context, the aim of this study was to describe the short-term clinical and sonographic evolution observed after a standardized ultrasound-based diagnostic and interventional pathway combining SSNB and corticosteroid-assisted capsular HD in patients with refractory AC.

## 2 Materials and methods

### 2.1 Study design and patient selection

This study was a retrospective observational analysis of patients treated in a tertiary musculoskeletal interventional unit. Data were obtained from a prospectively maintained clinical database routinely used in our institution for clinical and quality-assurance purposes. The diagnostic and interventional pathway formed part of a standard clinical care and was not originally designed as a research protocol. Accordingly, the present analysis was conceived as an exploratory description of clinical and sonographic outcomes observed after implementation of this standardized pathway rather than as an efficacy study.

For the purpose of this study, patients referred to our unit with suspected AC were identified through retrospective review of the database within the study period (December 2023 to April 2024). All patients underwent this standardized diagnostic assessment and were classified according to whether AC was confirmed or excluded. Only those with clinically and sonographically confirmed AC, fulfilling predefined criteria for interventional treatment, were included in the outcome analysis, whereas patients without confirmed AC were managed according to the alternative diagnosis.

All patients were evaluated at baseline, immediately before the interventional procedure, and at 3 months after treatment. Patients were also followed clinically for up to 1 year as part of routine care to identify documented symptom recurrence or persistent functional limitation. Detailed goniometric and ultrasound measurements were not systematically repeated at 12 months.

The clinical diagnosis of AC was based on the presence of progressive shoulder pain associated with global restriction of both active and passive ROM of the glenohumeral joint, particularly affecting external rotation. Typical clinical features included insidious onset of shoulder pain, frequently worsening at night, progressive stiffness and limitation in daily activities.

Physical examination findings considered compatible with AC included marked limitation of passive external rotation with the arm adducted, together with restriction of forward flexion and abduction. Internal rotation was also assessed. Range of motion was measured using a standard goniometer according to a predefined examination procedure. The same clinical team performed baseline and follow-up assessments. Assessors were not blinded to the assessment timepoint or treatment status, because measurements were obtained during routine clinical care.

Invasive treatment was performed only in patients in whom both clinical findings and ultrasound examination confirmed the diagnosis of AC, specifically demonstrating capsular thickening of the inferior glenohumeral recess (see section “Ultrasound assessment”). In addition, the procedure was indicated only in patients with refractory AC, defined as persistent shoulder pain and limitation of both active and passive ROM for at least 3 months despite conservative treatment, including analgesic medication, physiotherapy or previous intra-articular injections. This threshold was selected as it corresponds to the transition from acute to chronic pain and is commonly used in previous clinical studies to define failure of initial conservative management (7, 8, 17, 18, 24). Immediately before HD, patients underwent an ultrasound-guided SSNB as part of the standard procedural protocol.

Patients without clinical findings consistent with AC or with normal capsular thickness on ultrasound examination were not treated with HD and were subsequently managed according to the alternative diagnosis identified during clinical evaluation.

Patients were excluded from the analysis if they had a history of previous shoulder surgery or fracture.

All procedures were performed by the same clinical team to ensure procedural consistency and minimize operator-related variability.

The study was conducted in accordance with the principles of the Declaration of Helsinki. Approval from the institutional ethics committee was obtained prior to data collection and analysis, and all patients had previously provided informed consent for the procedure and for the use of their clinical data for research purposes.

The process of patient selection and diagnostic confirmation is illustrated in Figure 1.

**FIGURE 1:**
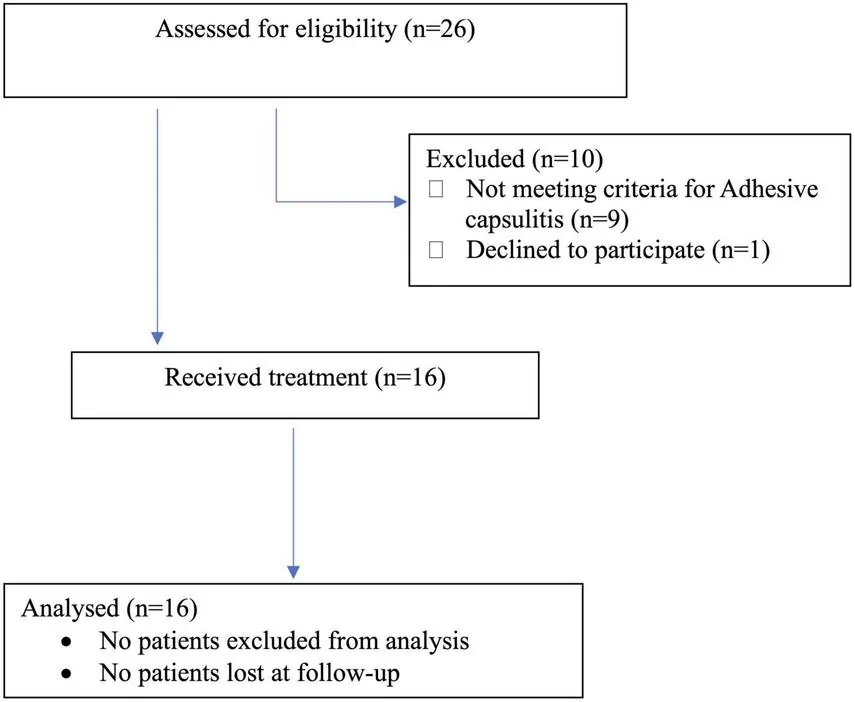
Flowchart of patient selection and diagnosis and treatment process.

### 2.2 Ultrasound assessment

All patients underwent ultrasound examination prior to the procedure using a Canon Aplio i800 ultrasound system equipped with a multifrequency linear transducer. All examinations and follow-up assessments were performed by the same experienced musculoskeletal ultrasound technician, following routine standardized clinical practice, with diagnosis confirmed by musculoskeletal ultrasound experts. Use of a single operator reduces interobserver variability; however, formal intra-rater and inter-rater reliability testing was not performed of the present cohort. The operator was not blinded to the assessment timepoint or clinical status.

A complete shoulder ultrasound examination was performed according to standard musculoskeletal imaging practice, including gray-scale and Doppler imaging and both static and dynamic evaluation of the rotator cuff, subacromial space, long head of the biceps tendon and glenohumeral joint.

Capsular thickness of the glenohumeral joint was assessed with the patient in the supine position and the transducer placed in the axillary region with the arm in slight abduction, which provides optimal exposure of the inferior capsular recess. The axillary recess was selected for measurement because capsular thickening in AC most frequently involves the inferior glenohumeral capsule, and provides a reliable and reproducible landmark for ultrasound assessment of capsular thickness (10–12). Although several ultrasound findings have been described in association with adhesive capsulitis, including synovial hypertrophy, hypervascularity on Doppler imaging and thickening of the coracohumeral ligament, capsular thickening of the inferior glenohumeral recess has been consistently reported as one of the most characteristic and reproducible imaging findings of frozen shoulder (10–12, 25, 26). For this reason, capsular thickness was used as the primary ultrasound parameter to support the clinical diagnosis of AC within our routine diagnostic pathway.

Capsular thickness was defined as the distance between the humeral cortical surface and the outer margin of the glenohumeral joint capsule at the inferior axillary recess, including the capsular-synovial complex. Measurements were obtained in both transverse and longitudinal planes: the humeral head was assessed in the transverse plane, while the humeral head-neck junction was evaluated longitudinally. Because capsular thickening may be heterogeneous, the maximum measured thickness was recorded for analysis. Ultrasound was not used as a standalone diagnostic test. A value >0.35 cm was used as a predefined supportive imaging criterion on the basis of previous ultrasound studies reporting pathological thickening of the inferior glenohumeral capsule and axillary recess in AC (13, 14). Although this threshold has been reported as suggestive of AC, it was not considered diagnostic in isolation. Given the overlap between affected and asymptomatic shoulders reported in the literature, this threshold was interpreted only in conjunction with the characteristic clinical pattern and, when available, side-to-side comparison.

### 2.3 Ultrasound-guided technique

All procedures were performed in a dedicated musculoskeletal interventional suite under strict aseptic condition. The intervention field was prepared using sterile drapes, gloves, gauze, ultrasound probe covers and sterile ultrasound gel. Skin antisepsis was performed using a chlorhexidine-based antiseptic solution according to current infection prevention recommendations.

All materials used during the procedure were sterile, including syringes, needles, extension tubing and dressings. A 25-gauge needle was used for subcutaneous local anesthesia, while 22-gauge 3.5-inch spinal needles were used for the ultrasound-guided interventions. Local anesthetic was prepared using a 5-ml syringe, whereas 10-ml and 20-ml syringes were used for the suprascapular nerve block and capsular HD injections, respectively. An extension tube was connected to the syringe during HD to facilitate controlled hydrostatic injection. At the end of the procedure, a sterile adhesive dressing was applied at the puncture site.

### 2.4 Ultrasound-guided suprascapular nerve block technique

After sterilization of the intervention area and the patient, the suprascapular nerve block was performed under ultrasound guidance using a posterior approach to the suprascapular notch. With the patient in a seated position, the suprascapular notch was identified using a high-frequency linear transducer, visualizing the suprascapular ligament and the suprascapular nerve beneath it. Color Doppler ultrasound was used to identify the suprascapular artery in order to avoid vascular puncture.

After subcutaneous local anesthesia using a 25-gauge needle and 2% lidocaine, a 22-gauge spinal needle (3.5-inch) was advanced under real-time ultrasound guidance toward the suprascapular notch (from lateral to medial) until the tip of the needle was positioned adjacent to the suprascapular nerve, just beyond the suprascapular ligament. Aspiration was performed prior to injection to exclude intravascular placement.

A mixture of 9 ml consisting of 4 ml of 2% lidocaine, 4 ml of 0.25% bupivacaine and 1 ml (40 mg) of depot triamcinolone was then injected around the nerve. The perineural corticosteroid component formed part of the institutional protocol used and was intended to prolong the anti-inflammatory and analgesic effect of the block.

### 2.5 Ultrasound-guided capsular hydrodilatation technique

Capsular HD was subsequently performed under ultrasound guidance using a posterior approach to the glenohumeral joint. The same skin entry point previously used for the suprascapular nerve block was utilized in order to minimize tissue trauma and avoid the need for an additional puncture and further subcutaneous local anesthetic infiltration.

With the patient in a seated position, the posterior glenohumeral joint recess was identified using ultrasound, placing the transducer over the posterior aspect of the shoulder, inferior to the posterior acromial margin, allowing visualization of the humeral head, posterior labrum and joint capsule.

Though the same entry point previously anesthetized, a 22-gauge spinal needle (3.5-inch) was advanced under real-time ultrasound guidance from lateral to medial (to avoid labrum injury) into the glenohumeral joint cavity. Correct intra-articular needle placement was confirmed by direct visualization of the needle tip within the joint space.

Hydrodilatation was performed by injecting a mixture composed of: 1 ml (40 mg) triamcinolone, 4 ml bupivacaine 0.25%, 4 ml lidocaine 2%, 11 ml 0.9% normal saline.

The solution was injected progressively under hydrostatic pressure in order to mechanically distend the contracted joint capsule. The target volume was 20 ml; injection was performed under continuous ultrasound monitoring and according to tissue resistance and procedural tolerance rather than by direct pressure measurement.

During injection, progressive resistance to fluid administration was typically encountered due to capsular stiffness. In all treated patients, a sudden decrease in injection resistance accompanied by dispersion of fluid beyond the capsular boundaries was noted, frequently accompanied by a subtle palpable pressure drop, suggesting capsular rupture. After this point, the injected solution flowed more easily into the joint space. Ultrasound visualization simultaneously showed progressive distension of the glenohumeral recess during fluid injection. Pressure was not directly quantified. The protocol did not compare capsule-preserving and capsule-rupturing techniques, and therefore no inference regarding the therapeutic contribution of rupture can be made.

To facilitate controlled injection and generation of sufficient hydrostatic pressure, the syringe was connected to an extension tube, allowing the operator to maintain stable ultrasound visualization while an assistant performed the injection.

After the procedures, patients were observed for a short period and discharged on the same day.

### 2.6 Outcome measures

Clinical outcomes were assessed by evaluating pain intensity, shoulder ROM and ultrasound-measured capsular thickness before the procedure and at 3 months. The 3-months timepoint was selected to describe short-term clinical and sonographic evolution after the intervention. All patients completed routine clinical follow-up at 12 months, either through outpatient visits or telephone contact. At this timepoint, follow-up focused on the presence of recurrent pain or shoulder stiffness suggestive of AC recurrence. Standardized functional examination and ultrasound reassessment were not routinely performed in asymptomatic patients and were reserved for those reporting recurrent symptoms or clinical deterioration. No patient fulfilled these criteria during follow-up, except for one patient with persistent residual symptoms attributed to concomitant calcific tendinopathy, who remained under routine clinical care.

Pain intensity was assessed using a visual analogue scale (VAS) ranging from 0 (no pain) to 10 (worst imaginable pain). A reduction of ≥2 points on the VAS was considered clinically meaningful improvement consistent with previously reported minimal clinically important differences for musculoskeletal pain outcomes (26).

Shoulder mobility was evaluated by measuring passive ROM using a standard goniometer according to a predefined examination procedure at baseline and follow-up by the treating clinical team. Measurements included forward flexion (0–180°), abduction (0–180°) and external (0–90) and internal rotation (0–90). Active ROM restriction was assessed as part of the diagnostic evaluation but was not retained as a longitudinal outcome measure. Formal intra-rater reliability was not evaluated in this cohort, and assessors were not blinded.

External rotation was measured with the arm adducted and the elbow flexed at 90°, with the arm positioned against the body. Internal rotation was measured with the patient in the supine position with the shoulder abducted and the elbow flexed at 90°, while the forearm was moved toward the examination table until maximal internal rotation was reached. In patients unable to achieve 90° abduction, internal rotation was measured at the maximum tolerated abduction angle. This approach follows standard clinical assessment methods for shoulder ROM (27).

An increase of at least 15° in ROM was considered clinically relevant improvement, as this threshold exceeds the expected measurement error of goniometric shoulder assessment reported in reliability studies (28).

No inferential subgroup analyses were retained in the revised analysis because the resulting groups were too small to support statistically meaningful comparisons.

### 2.7 Statistical analysis

Statistical analysis was performed using SPSS statistical software for Windows, version 26.0. Continuous variables were expressed as mean ± standard deviation, while categorical variables were presented as frequencies and percentages.

Within-patient changes in pain intensity, capsular thickness, and shoulder mobility between baseline and 3 months were evaluated using paired Student’s *t*-tests. Given the limited sample size and the potential sensitivity of parametric analyses to departures from distributional assumptions, Wilcoxon signed-rank tests were additionally performed as complementary non-parametric analyses. Findings were interpreted considering the concordance in the direction of change and statistical results across both paired analytical approaches. Mean within-patient changes and corresponding 95% confidence intervals were calculated, where appropriate, to describe the magnitude and precision of the observed differences. The proportion of patients achieving a >50% reduction in pain from baseline was also estimated. A two-sided *p*-value < 0.05 was used as the threshold for statistical significance.

Given the limited sample size and the potential sensitivity of parametric methods to violations of assumptions in small samples, the analysis was complemented with a bootstrap resampling simulation (1000 replicates). This approach allows a more robust empirical estimation of the distribution of mean differences, standard errors, and confidence intervals, improving the reliability of the results in contexts where normality or homoscedasticity cannot be fully guaranteed (29–31). *P*-values from bootstrap resampling were derived from the proportion of resampled distributions crossing zero.

No inferential subgroup analyses according to comorbidity status were performed because the resulting subgroup sizes were considered insufficient to support reliable statistical comparisons. Standardized effect-size estimates were not retained in the revised analysis because their magnitude may be unstable and potentially inflated in a small sample; therefore, the interpretation of the observed changes was based primarily on absolute within-patient differences and their corresponding confidence intervals.

No formal adjustment for multiple comparisons was applied because of the exploratory and hypothesis-generating nature of the study. Accordingly, *p*-values were interpreted cautiously and in conjunction with the magnitude, precision, direction, and consistency of the observed changes across related clinical and sonographic outcomes.

## 3 Results

### 3.1 Patient flow and diagnostic confirmation

A total of 26 patients referred to our musculoskeletal interventional unit with a suspected diagnosis of AC were considered for inclusion in the analysis. One patient declined evaluation and treatment and was therefore excluded from the database and posterior analysis. Agreement between the referring diagnosis and the final diagnosis established after specialized clinical and ultrasound evaluation was low. Of the 25 evaluable patients, the diagnosis was confirmed in 16 individuals (64%) and excluded in 9 (36%) (Table 1).

**TABLE 1:** Agreement table.

Differential diagnosis after specialized evaluation							
		Capsulitis	Soft-tissue fibrosis (lipomatosis)	Biceps tenosynovitis	Trapezius myofascial syndrome	Trapezius and pectoralis major myofascial syndrome	Supraspinatus calcific tendinopathy with tendon tear
Referral diagnosis	Capsulitis	16	1	1	5	1	1

### 3.2 Baseline characteristics

The demographic and clinical characteristics of the treated cohort (*n* = 16) are summarized in Table 2.

**TABLE 2:** Demographic and clinical characteristics of the treated cohort (*n* = 16).

Gender	*N* (%)
Female	10 (62.5%)
Male	6 (37.5%)
Age	51.2 (9.8)
Affected shoulder:	
Left	11 (68.8%)
Right	5 (31.3%)
Symptom duration (months)	7.8 (4.2)
Previous shoulder injury before capsulitis	
Yes (secondary forms)	13 (81%)
No (primary forms)	3 (19%)
Previous shoulder injury type	
Prolonged positioning-related injury (during a cholecystectomy)	1 (7.7%)
Bankart and Hill Sachs lesions	1 (7.7%)
Long head of the biceps tendon rupture	1 (7.7%)
Long head of the biceps tenosynovitis	1 (7.7%)
Acute supraspinatus tendinosis	4 (30.7%)
Calcific tendinopathy of the supraspinatus	5 (38.5%)
Stage of adhesive capsulitis	
1	2 (12.5%)
2	8 (50%)
3	6 (37.5%)
All patients had previously received NSAIDs	
Previous oral corticosteroids	
Yes	4 (25%)
No	12 (75%)
Previous Physiotherapy	
Yes	12 (75%)
No	4 (25%)
Previous intraarticular infiltration	
Yes	5 (31%)
No	11 (69%)
None of them received previous hydrodilatation	

The use of regular medications was not systematically recorded in this study. However, based on retrospective review of clinical records, none of the patients were receiving strong opioids, high-dose weak opioids, or systemic corticosteroids at the time of the procedure. In addition, no documented changes in analgesic medication had been made during the 8 weeks preceding the intervention.

### 3.3 Clinical outcomes

The mean reduction in pain intensity measured by the VAS was 6.69 ± 1.67 points, with a standard error of 0.42 and a 95% confidence interval ranging from 5.8 to 7.6. Fifteen of 16 patients (93.8%) experienced a >50% reduction in pain from baseline (Table 3).

**TABLE 3:** Changes in the evaluated variables (entire adhesive capsulitis group).

	Healthy shoulder	Pre	Post	t de Student	Z-Wilcoxon	% patients with an improvement >50%
Visual analogic scale PAIN (0–10)	N/A	7.81 (0.91)	1.13 (1.75)	16.09 (*p* < 0.001)	**−**3.54 (*p* < 0.001)	93.8%
Sagittal neck capsular thickness (cm) (*n* = 15)	0.17 (0.04)	0.57 (0.29)	0.17 (0.04)	4.92 (*p* < 0.001)	**−**3.41 (*p* < 0.001)	
Sagittal head capsular thickness (cm) (*n* = 14)	0.16 (0.03)	0.52 (0.26)	0.18 (0.06)	4.3 (*p* = 0.001)	**−**3.30 (*p* = 0.001)	
Transversal head capsular thickness (cm) (*n* = 15)	0.17 (0.03)	0.53 (0.27)	0.18 (0.08)	4.64 (*p* < 0.001)	**−**3.35 (*p* = 0.001)	
Flexion grades (0–180)	N/A	79.06 (23.04)	171.56 (22.56)*	**−**15.02 (*p* < 0.001)	**−**3.53 (*p* < 0.001)	
Abduction grades (0–180)	N/A	66.88 (22.235)	170.31 (22.32)*	**−**13.85 (*p* < 0.001)	**−**3.52 (*p* < 0.001)	
Internal rotation grades (0–90)	N/A	73.13 (14.93)	79.38 (13.40)*	**−**2.18 (*p* = 0.046)	**−**2.04 (*p* = 0.04)	
External rotation grades (0–90)	N/A	15.31 (8.85)	80.31 (14.43)*	**−**14.61 (*p* < 0.001)	**−**3.53 (*p* < 0.001)	

The asterisk indicates values for which the mean ± standard deviation mathematically exceeds the physiological maximum range of motion; this reflects statistical dispersion and does not represent actual values beyond the physiological range, as explained in the text.

Bootstrap resampling yielded a similar estimated mean change (6.69 points), with minimal bias (0.013), a standard error of 0.40 and a 95% confidence interval of 5.9 and 7.4. These sensitivity analyses supported the stability of the estimated within-patient change in this cohort.

Given the exploratory design and small sample size, no correlation analyses between baseline and post-intervention pain scores were retained in the revised analysis.

Post-treatment flexion and abduction values approached the upper physiological limit in several patient. The dispersion of post-treatment values interindividual variability, with some patients achieving full ROM and others retaining residual limitation. Values exceeding physiological limits are therefore a statistical artifact rather than a true clinical finding.

### 3.4 Changes in capsular thickness

Capsular thickness in the affected shoulder was significantly greater than in the contralateral asymptomatic shoulder at all measurement levels (sagittal neck *p* = 0.0026; sagittal head *p* = 0.0033; transverse head *p* = 0.0013). These comparisons support the presence of side-to-side structural asymmetry but should be interpreted cautiously because of the small sample and multiple related measurements.

Capsular thickness decreased at 3 months across all ultrasound measurement planes. At the sagittal neck, mean thickness decreased by 0.39 cm ± 0.31, (95% CI 0.22–0.57 cm). Comparable mean reduction were observed at the sagittal head (0.33 ± 0.29 cm) and transverse head (0.34 cm ± 0.28 cm) (Table 3). Bootstrap sensitivity analyses produced similar estimates.

These findings demonstrate a measurable reduction in ultrasound-assessed capsular thickness after the intervention. They should not be interpreted as histological normalization of the capsule or as proof that the intervention caused structural remodeling.

Missing ultrasound measurements were due to technical limitations or incomplete image acquisition in some patients.

### 3.5 Improvements in shoulder range of motion

At 3 months, forward flexion had increased by a mean of 92.5° (95% CI 79.38–105.62°) compared with baseline.

Mean abduction improved by 103.44° over the same period, while external rotation improved by a mean of 65°.

In contrast, internal rotation showed a smaller mean increase of 6.25°. The associated *p*-value was exploratory (*p* = 0.046 by paired *t*-test; *p* = 0.04 by Wilcoxon signed-rank test) and should be interpreted cautiously in the context of multiple comparisons.

Bootstrap sensitivity analyses yielded estimates consistent with the direction of the observed changes in ROM.

### 3.6 Exploratory subgroup analyses

The original exploratory analyses according to comorbidity status were removed because subgroup sizes of three to eight patients were insufficient for reliable inferential comparison.

## 4 Discussion

This observational study describes the clinical and sonographic outcomes of patients with refractory AC managed through a standardized pathway integrating structured clinical assessment, ultrasound confirmation, SSNB, and corticosteroid-assisted glenohumeral HD. Three main findings emerged. First, structured clinical and ultrasound reassessment did not confirm AC in more than one-third of patients referred with this diagnosis. Second, patients with confirmed refractory AC showed substantial and clinically meaningful improvements in pain and shoulder mobility at 3 months. Third, these clinical changes occurred in parallel with a measurable reduction in ultrasound-assessed inferior glenohumeral capsular thickness. Taken together, these findings support the clinical relevance of a standardized diagnostic and interventional pathway and provide preliminary evidence of parallel clinical and sonographic evolution after treatment.

Given the observational uncontrolled design, the contribution of the intervention cannot be separated from natural disease evolution, regression to the mean, contextual effects, or concomitant rehabilitation. Accordingly, the magnitude of the observed changes should not be interpreted as a causal treatment-effect estimate. Nevertheless, the consistency and magnitude of the changes across pain, multiple planes of shoulder motion, and ultrasound-assessed capsular thickness are noteworthy in a population with persistent symptoms despite previous conservative treatment.

One of the most clinically relevant observations was that 9 of 25 patients referred with suspected AC did not meet the predefined clinical and sonographic criteria after structured evaluation. Although the sample is too small to estimate diagnostic accuracy of the pathway, this finding illustrates the heterogeneity of patients referred under the label of frozen shoulder and is consistent with the diagnostic challenges previously described in AC (1, 2, 7–9). In this cohort, ultrasound complemented rather than replaced clinical assessment and was used both to characterize capsular involvement and to identify alternative shoulder pathology. This integrated assessment may be particularly relevant before invasive treatment, as improved diagnostic confidence could contribute to more appropriate patient selection.

Adhesive capsulitis remains primarily a clinical diagnosis, and no single ultrasound measurement should be considered diagnostic in isolation. Capsular thickening of the inferior glenohumeral recess is among the most consistently reported ultrasound findings in AC (10–12, 32). Nevertheless, published measurements and proposed cut-off values vary according to acquisition technique and anatomical measurement site. The >0.35 cm threshold used in our diagnostic pathway was therefore considered a predefined supportive imaging criterion rather than a standalone diagnostic boundary. Previous studies have also demonstrated good correlation between ultrasound measurements of axillary recess capsular thickness and MRI findings, supporting ultrasound as a reliable method for assessing capsular thickening in AC (14). Although previous ultrasound studies have supported the diagnostic relevance of increased inferior capsular thickness, they have also demonstrated partial overlap in capsular thickness measurements between affected and asymptomatic shoulders (10–14, 33). Therefore, the >0.35 cm threshold should be regarded as a supportive imaging criterion rather than a definitive diagnostic cutoff. In routine practice, comparison with the contralateral shoulder and integration with the overall clinical and sonographic findings are likely to be more informative than reliance on a single absolute threshold. Accordingly, in our study, ultrasound findings were interpreted in conjunction with clinical features and contralateral comparison. However, formal validation of this threshold within our specific acquisition protocol and operator-specific reliability testing were beyond the scope of the present retrospective analysis.

At 3 months, the cohort showed large absolute changes in pain and shoulder mobility. The mean reduction in pain intensity was 6.69 points on the VAS, and 15 of 16 patients achieved a reduction greater than 50% from baseline. Improvements in ROM were particularly marked for forward flexion, abduction, and external rotation, with mean increases 92.5°, 103.44° and 65°, respectively. External rotation is particularly relevant because restriction in this plane is considered one of the most characteristic features of AC (8, 18). The magnitude of the observed changes exceeded the predefined thresholds for clinical relevance and showed a consistent direction across paired parametric, non-parametric, and bootstrap analyses. Although the uncontrolled design prevents attribution of these improvements exclusively to the intervention, the extent and consistency of the observed changes are clinically notable, particularly considering the persistent symptoms and previous conservative treatment of the included population. Internal rotation showed a smaller mean change, probable reflecting the comparatively limited baseline impairment in this plane of motion.

The reported mean ± standard deviation describes the distribution of the measurements in ROM rather than the observed extremes. Consequently, although the mathematical upper bound of the descriptive statistics may appear to exceed conventional physiological limits, no individual patient achieved shoulder motion beyond the normal physiological range. These apparent values therefore represent a statistical artifact of data presentation rather than a true clinical finding.

Follow-up ultrasound demonstrated a consistent reduction in inferior glenohumeral capsular thickness across all evaluated measurement planes. We deliberately avoid describing this finding as capsular “normalization,” because capsular and synovial thickness on imaging may reflect several biological components, including inflammation, synovial change, edema, extracellular matrix deposition, and fibrosis. Therefore, the observed reduction may reflect resolution of inflammatory or edematous components, progressive capsular remodeling, or a combination of these mechanisms. Measurement variability cannot be excluded, particularly in the absence of blinded assessment and formal operator-specific reliability testing. However, the consistency of the reduction across the three ultrasound measurement planes, together with the parallel improvement in pain and shoulder mobility, supports the biological plausibility of a genuine change in capsular involvement over time.

These findings do not establish histological reversal of fibrosis but suggest that ultrasound-assessed capsular thickness may be responsive to clinical evolution in AC. This observation is particularly relevant because most interventional studies have focused predominantly on symptomatic and functional outcomes, whereas longitudinal imaging changes after treatment remain less well characterized. Serial ultrasound assessment of the inferior capsule therefore deserves further evaluation as a potential imaging marker of disease evolution and treatment response. Prospective studies incorporating blinded ultrasound assessment, formal intra- and inter-rater reliability testing, and correlation with MRI or other reference imaging techniques are required to clarify the biological and clinical significance of these longitudinal changes.

The literature on HD is heterogeneous with respect to patient selection, injection approach, injectate composition, volume, and the intended preservation or rupture of the capsule (15–17, 20, 34). Such procedural variability may contribute to the inconsistent outcomes reported across studies. In our protocol, a 20 ml injection volume was selected based on literature and clinical experience to balance capsular distension and patient tolerance (15–17). Injection was performed progressively under ultrasound monitoring and according to tissue resistance and patient tolerance rather than direct pressure measurement.

In all treated patients, a sudden loss of injection resistance and extra-capsular fluid dispersion was observed and considered sonographically compatible with capsular rupture. Pressure was not directly quantified, and the present study was not designed to compare capsule-preserving and capsule-rupturing techniques. Whether capsular rupture represents a desirable therapeutic endpoint remains uncertain. Recent comparative data suggest that preservation and rupture of the capsule may be associated with different clinical outcomes, emphasizing the need for greater procedural standardization and further comparative investigation (34). Accordingly, the present findings cannot establish the therapeutic contribution of presumed capsular rupture.

The present study evaluated the combined protocol as it was used in routine care and cannot determine whether HD itself, SSNB, corticosteroid exposure, capsular rupture, or their interaction accounted for the observed evolution. However, the bundled nature of the intervention was deliberate rather than incidental. The protocol was designed to address complementary mechanisms involved in AC: nociceptive input through SSNB, inflammatory activity through corticosteroid administration, and capsular restriction through mechanical distension and probable capsular rupture. SSNB has previously demonstrated efficacy in reducing pain and facilitating functional recovery in AC, and its use in combination with HD has also been described (18, 21–23, 35, 36). From this perspective, the present findings should be interpreted as outcomes of an integrated interventional strategy rather than as evidence for any individual procedural component. Comparative studies using multi-arm or factorial designs will be required to determine the relative contribution of each element.

The total triamcinolone dose was 80 mg, divided between the SSNB (40 mg) and intra-articular HD injectate (40 mg). This reflected our institutional protocol and the use of two distinct anatomical targets. Corticosteroid administration during HD is well established in the literature, while corticosteroid-containing SSNB protocols have also been described (15–17, 21–23, 35). Nevertheless, the present study provides no evidence that this total dose or its distribution is optimal. The total corticosteroid dose reflected our institutional protocol rather than evidence of superiority over alternative regimens. The dose was divided because the SSNB an intra-articular HD were intended to target different anatomical structures and achieve complementary therapeutic effects, rather than increasing corticosteroid exposure at a single site. Cumulative corticosteroid exposure and potential systemic or local adverse effects should be considered when applying such a protocol, particularly in patients with diabetes or other corticosteroid-sensitive conditions. No major or documented corticosteroid-related adverse events occurred in this cohort, although the sample is insufficient for robust safety conclusions.

The predominance of secondary AC (81%) is also relevant to interpretation. Importantly, our unit receives patients with AC from the entire hospital healthcare area rather than exclusively highly selected secondary cases. Therefore, the high proportion of secondary AC observed in this cohort may partly reflect the case mix encountered in routine clinical practice within our catchment population rather than referral enrichment alone. Although secondary forms may differ from primary idiopathic AC in pathophysiology, prognosis, and treatment response, their predominance also increases the real-world relevance of the cohort for clinicians managing refractory AC associated with concomitant shoulder pathology. Accordingly, extrapolation of the present findings to predominantly idiopathic AC populations should be cautious.

The heterogeneity of previous conservative treatment should also be considered. Patients had received different combinations of NSAIDs, oral corticosteroids, physiotherapy, and previous intra-articular injections before referral. However, all included patients had persistent pain and restriction of active and passive ROM despite previous conservative management and fulfilled the predefined criteria for refractory AC before undergoing the interventional pathway. Furthermore, retrospective review did not identify changes in analgesic medication during the 8 weeks preceding the procedure, and none of the patients were receiving strong opioids, high-dose weak opioids, or systemic corticosteroids at the time of treatment. Although prior treatment heterogeneity remains a potential source of confounding, the cohort represents the type of clinically heterogeneous refractory population commonly encountered in real-world musculoskeletal practice.

No major procedural complications were documented. Mild transient post-procedural discomfort resolved spontaneously within the first day. All patients completed routine clinical follow-up at 12 months, either through outpatient review or telephone contact. No patient developed recurrent pain or shoulder stiffness suggestive of AC requiring further clinical evaluation or treatment. One patient remained under routine clinical follow-up because of persistent symptoms attributable to concomitant calcific tendinopathy rather than recurrent AC. These longer-term observations should be interpreted descriptively, as standardized 12-months ROM and ultrasound assessments were not routinely performed in asymptomatic patients but were reserved for those with suspected clinical recurrence. Consequently, the absence of documented recurrence reflects routine clinical follow-up rather than a protocolized imaging or functional reassessment. Accordingly, recurrence was pragmatically defined as the reappearance of pain and/or shoulder stiffness requiring further clinical evaluation or treatment. Nevertheless, these observations provide preliminary descriptive information on the clinical course of this cohort beyond the predefined 3-months outcome assessment.

Several limitations should be acknowledged. First, the retrospective, uncontrolled observational design precludes causal inference and does not allow the observed changes to be distinguished from natural history, regression to the mean, placebo or contextual effects, or concomitant rehabilitation. Second, the small sample size limits the precision and generalizability of the estimates. For this reason, subgroup analyses were removed from the revised analysis, and interpretation focuses on absolute within-patient changes, confidence intervals, and the consistency of findings across analytical approaches rather than on comparisons between small clinical subgroups. No formal adjustment for multiple comparisons was applied because of the exploratory nature of the analysis; therefore, individual *p*-values, particularly for marginal findings, should be interpreted cautiously and in the context of the magnitude, direction, and consistency of the observed changes.

Third, ultrasound and ROM assessments were obtained during routine clinical care and were not blinded, and formal operator-specific reliability testing was not available. Although the same experienced clinical team and ultrasound operator performed serial assessments using standardized examination and acquisition procedures, observer-related measurement bias cannot be excluded. In addition, the >0.35-cm capsular thickness threshold was used as a supportive imaging criterion within a combined clinical and ultrasound diagnostic pathway and was not independently validated for the specific acquisition protocol used in this cohort. The combined nature of the intervention also prevents determination of the individual contribution of SSNB, corticosteroids, HD, and probable capsular rupture. Finally, most patients had secondary AC and had received heterogeneous previous conservative treatments, and standardized goniometric and ultrasound assessment was limited to 3 months. Routine clinical follow-up extended to 12 months but did not include systematic long-term imaging or ROM measurements.

These limitations notwithstanding, the use of a standardized diagnostic pathway, predefined interventional protocol, serial within-patient assessment, and concordant changes across pain, several planes of shoulder mobility, and ultrasound-assessed capsular thickness provides a consistent preliminary dataset in a refractory real-world AC population. The present findings do not establish therapeutic efficacy or the independent contribution of individual procedural components. However, they support further prospective controlled evaluation of an integrated ultrasound-guided diagnostic and interventional strategy and highlight the potential value of serial ultrasound assessment for characterizing structural evolution in AC.

Future studies should aim to validate this protocol in larger prospective controlled cohorts, to clarify the individual contribution of each component, and to explore the potential role of ultrasound-derived parameters as biomarkers for treatment response and disease monitoring.

## Data Availability

The raw data supporting the conclusions of this article will be made available by the authors, without undue reservation.
